# Total flavonoids of *Abrus cantoniensis* inhibit CD14/TLR4/NF-κB/MAPK pathway expression and improve gut microbiota disorders to reduce lipopolysaccharide-induced mastitis in mice

**DOI:** 10.3389/fmicb.2022.985529

**Published:** 2022-08-24

**Authors:** Wen-Jing Sun, En-Yun Wu, Ge-Yin Zhang, Bai-Chang Xu, Xiao-Gang Chen, Kai-Yuan Hao, Ying Wang, Ling-Zhi He, Qi-Zhuang Lv

**Affiliations:** ^1^Guangxi Key Laboratory of Agricultural Resources Chemistry and Biotechnology, College of Biology and Pharmacy, Yulin Normal University, Yulin, Guangxi, China; ^2^State Key Laboratory for Conservation and Utilization of Subtropical Agro-bioresources, College of Animal Science and Technology, Guangxi University, Nanning, China

**Keywords:** *Abrus cantoniensis*, flavonoids, LPS, mastitis, mRNA, blood-milk barrier, gut microbiota

## Abstract

Established a model of lipopolysaccharide (LPS)-induced mastitis in mice, pathological sections and myeloperoxidase were used to detect the degree of tissue damage, enzyme-linked immunosorbent assay (ELISA) was performed to detect the expression of pro-inflammatory cytokines, meanwhile fluorescence quantitative PCR experiments were performed to detect the mRNA expression of CD14/TLR4/NF-κB/MAPK signalling pathway, and the faeces of mice were collected for 16S measurement of flora. The results showed that *Abrus cantoniensis* total flavonoids (ATF) could significantly reduce the damage of LPS on mammary tissue in mice and inhibit the secretion of inflammatory factors such as TNF-α, IL-1β and IL-6. At the mRNA level, ATF inhibited the expression of CD14/TLR4/NF-κB/MAPK pathway and enhanced the expression of tight junction proteins in the blood-milk barrier. In the results of the intestinal flora assay, ATF were found to be able to regulate the relative abundance of the dominant flora from the phylum level to the genus level, restoring LPS-induced gut microbial dysbiosis. In summary, ATF attenuated the inflammatory response of LPS on mouse mammary gland by inhibiting the expression of CD14/TLR4/NF-κB/MAPK pathway, enhancing the expression of tight junction proteins and restoring LPS-induced gut microbial dysbiosis. This suggests that ATF could be a potential herbal remedy for mastitis.

## Introduction

Mastitis is defined by the presence of hard, swollen, hot, aching breasts, ill cattle and sick sheep refusing to feed young animals or milking artificially (Pang et al., 2014). Common to postpartum lactation, this disease is of higher incidence, and mastitis in dairy goats is most likely to develop 1–20 days after delivery of lambs (Lv, 2019; Liang and Shuhua, 2020). Traditional Chinese medicine (TCM) therapy, which is a traditional treatment in China with many kinds of advantages such as low toxicity, few residues and economic applicability (Zhang, 2019), is the current hot direction in the field of prevention and treatment of mastitis in dairy cattle. China is the home country of Chinese herbal medicine, with abundant resources in TCM, and more than a thousand TCMs have been applied in the clinic (Tian, 2006; Zeng, 2020). TCM can play a role in the prevention and treatment of mastitis in dairy cattle by supplementing nutrition, enhancing body immunity as well as antibacterial and anti-inflammatory effects (Li, 2020). One of the most common pathogens of clinical mastitis in cows is Escherichia coli, which causes mastitis in cows that are usually single-lactation, with hardened udders, lumps and greyish-yellow milk with a foul odour (Zhang, 2019).

LPS, as an important component of the cell wall of Escherichia coli, is a classical endotoxin. When LPS enters the host organism, it stimulates the production of a variety of cytokines, such as TNF-α and IL-1β, by effector cells (Wu, 2019). Abundant expression of cytokines stimulates neutrophil recruitment to the lesion location, which in turn exerts neutrophil clearance (Guan, 2020). In the field of mastitis research, the symptoms of LPS-stimulated mastitis in mice are the same as those of mastitis in cows in clinical cases (Yu et al., 2020), so it can be used to investigate the mechanism of mastitis and to screen drugs for prevention and treatment. TLR4 is a transmembrane receptor for LPS of Gram-negative bacteria (Miyake, 2004). It has been found that the anti-inflammatory effect of most herbs on the LPS-mediated TLR4 signalling pathway is achieved by affecting the transcription of NF-κB and p65 transcription factors and controlling the release of associated inflammatory factors. Therefore, a number of anti-inflammatory drugs, including TCM components, are currently being studied with the LPS-TLR4 signalling pathway as their target (Huakuang et al., 2011).

CD14 is a high-affinity receptor for the LPS and LPS-binding protein (LBP) complexes (Wang et al., 2019). It recognises and binds LPS, causing cellular tyrosine phosphorylation, nuclear factor NF-κB translocation, triggering cytokine release and oxygen free radical production, and plays an important role in a range of responses induced by the immune and defence systems (Yan, 2020). The CD14 amplification response can be beneficial to the host by inducing an adequate inflammatory and immune response to eliminate invading microbes, but also detrimental to the host due to excessive inflammation and pathogen dissemination (Rong et al., 2017). Signal transduction of CD14 with LPS mainly includes transmembrane signal transduction and intracellular signal transduction. Among them, intracellular signalling would activate the corresponding NF-κB and mitogen-activated protein kinase (MAPK) pathways, which ultimately cause IL-1, IL-6 and TNF-α *Et al* release, leading to the occurrence of disease (Li et al., 2002).

The function of the blood-milk barrier is dependent on the integrity of the mammary epithelium and the way in which the cells connect to each other in concert (Wang, 2018). The connections between mammary epithelial cells are mainly composed of tight junctions, adhesion junctions and gap junctions, with tight junctions being the main connection between adjacent mammary epithelial cells and endothelial cells (Miessler et al., 2018). Intercellular tight junctions are a dynamic complex meshwork, and it has now been demonstrated that tight junctions are formed by a variety of tight junction proteins, including Occludin, Claudins, ZOs, Jams, as well as functional proteins, among others (Visser et al., 2009).

The gut microbiota is a huge and complex ecosystem (Tian et al., 2021), and when the balance is disturbed, it may affect the entire health of the organism (Butel, 2014). In recent years it has been found that the gut microbiota and the inflammatory response are closely related and that disturbances in the gut microbiota can lead to an enhanced inflammatory response (Vaiserman et al., 2017), which in turn can influence the type or abundance of the gut microbiota. It has been found that mastitis in mice leads to a disturbance in their intestinal microbiota, increasing the number of pathogenic bacteria and decreasing the number of beneficial flora in the gut (Guo et al., 2020). One study analysed fecal 16S rDNA diversity in 130 patients with acute pancreatitis and 35 healthy subjects, found that the structure of the gut microbiota was significantly different between the two study groups and was closely related to the systemic inflammatory response and impaired intestinal barrier and the abundance of beneficial bacteria such as blautia was significantly lower in severe patients than in mild and moderate patients (Lu et al., 2021).

*Abrus cantoniensis* is a member of the legume family Acaciaceae, also known as magnoflorine, red hen’s wort, yellow food grass, clover lepidotrichia, etc. Apart from the seeds, the whole plant can be used as medicine and food, mainly in Guangxi, Guangdong, Hunan and other provinces in China, with the effect of clearing heat and detoxifying the liver and relieving pain (Xu et al., 2005; Chen and Mo, 2008; Shan et al., 2011). Studies have shown that the whole plant of *A. cantoniensis* is rich in chemical components, mainly triterpenoids, flavonoids, polysaccharides, alkaloids and anthraquinones and other components, which are abundant in medicinal value (Yang et al., 2015). In this paper, we investigated the *A. cantoniensis* total flavonoids (ATF) effect on LPS-induced mastitis in mice and explored the ATF effect on the regulation of gut microbiota by 16S-rDNA sequencing. This may provide a theoretical basis for further exploration of the medicinal value of ATF.

## Materials and methods

### Materials and chemicals

The dried whole plant of *A. cantoniensis* (*Abri Herba*) was purchased in a local market (Nanning, China) [No.20200201]. Rutin standards (HPLC ≥ 98%) and LPS (HPLC ≥ 99%) were purchased at Beijing Solaibao Technology Co., Ltd. Absolute ethanol, sodium nitrite were purchased at Chengdu Cologne Chemical Co., Ltd., aluminium nitrate was purchased at Tianjin Da Mao chemical reagent factory, sodium hydroxide was purchased at Chengdu Jinshan Chemical Reagent Co., Ltd., and the above chemical reagents were analytical grade. Mouse IL-1β, IL-6, TNF-α and MPO ELISA kits were purchased from Jiangsu Jingmei industrial Co., Ltd. (Yancheng, Jiangsu, China). TRIzol reagent, First-strand cDNA Synthesis Mix and RealStar Green Fast Mixture were purchased from GenStar Co., Ltd. (Beijing, China). All other reagents in the experiments were purchased from China and were analytically pure.

### Animals

Kunming mice (6–7 weeks, 30–35 g, 20 male and 60 female) were procured from Tianqin Biotechnology Co., Ltd. in Changsha, China. Mice were adapted for 1 week in free light, at 22 ± 2°C and 50 ± 5% relative humidity, at the same time as being given adequate standard food and water (Chen et al., 2021). After 1 week, cages were randomly combined in a male to female ratio of 3:1 in order to obtain pregnant females. All animal experimental protocols for this experiment were approved by the institutional animal care and use Committee of Guangxi University (Nanning, China) [No. Gxu-2021-145] and performed in accordance with the guide for the care and use of laboratory animals of the National Institutes of Health.

### Extraction and purification of ATF

The ultrasound-assisted extraction was carried out according to the procedure in the previous study(Wu et al., 2022). Briefly, 47 ml of 50% ethanol solution was added for every 1 g of *A. cantoniensis* powder according to the material-to-liquid ratio of 1:47, and the extraction was cycled 4 times at an ultrasonic power of 125 W and an ultrasonic time of 40 min. The flavonoids were extracted from the solution when the solution changed from clarified to brownish yellow after the dried and crushed fragments of *A cantoniensis* were soaked in ethanol solution, and the analysis of its composition has been reported extensively(Yang et al., 2015; Yu, 2019). The extract was filtered and purified through D101 macroporous resin, the solution turned translucent yellow and the flavonoid content was determined using a rutin standard (*y* = 12.626*x* + 0.0023, *R*^2^ = 0.9997), which was dried and concentrated in a vacuum freeze dryer to obtain the light-yellow powder. Freeze-dried ATF in powder form was dissolved in 50% ethanol solution and its flavonoid content was determined using Rutin Standard Associate. The results showed that the purity of the ATF powder exceeded 76.7% at 25°C or room temperature.

### Building a mastitis model in mice

The ATF powdered was dissolved in sterilised water and prepared in three concentrations of 10 mg/ml, 50 mg/ml and 100 mg/ml. Dexamethasone was dissolved in sterilised water and prepared to 0.5 mg/ml. LPS was dissolved in saline and prepared to 0.2 mg/ml. The lactating mice, 7 ± 2 days after the birth of their offspring, were divided into four groups as follows:

- No-treatment (NT) group: gavage of 10 ml/kg sterile water without any treatment for 1 week as a vehicle control.
- Model control (MC) group: gavage of 10 ml/kg sterilised water for 1 week and injection of 50 μl 0.2 mg/ml LPS into breast tissue on the last day.
- Dexamethasone (DEX) group: gavage of 5 mg/kg dexamethasone solution for 1 week and injection of 50 μl 0.2 mg/ml LPS into breast tissue on the last day.
- ATF dose group: gavage of 100 mg/kg, 500 mg/kg and 1,000 mg/kg ATF for 1 week and mammary tissue injection of 50 μl 0.2 mg/ml LPS on the last day.

The specific steps are as follows (Table 1): Kunming mice were gavaged with 0.1 ml of sterilised water or drug solution per 10 g body weight for 1 week and the construction of the LPS-induced mastitis model in mice was started 1 h after the end of gavage on the 7th day. Mice were anaesthetised by intraperitoneal injection of chloral hydrate (35 mg of chloral hydrate powder in 1 ml of saline) and 50 μl of LPS (0.2 mg/ml) solution or saline was injected into two abdominal mammary glands to induce mastitis. After 12 h of model construction, gavage was performed again and changes in mouse status and mammary area were observed. 24 h after the injection of LPS, the mammary gland was collected for subsequent experiments.

**Table 1:** Different treatment groups in mice.

Group	Treatment	Duration
NT	Sterile water (gavage)	Days 1–7
MC	sterile water (gavage)	Days 1–7
	50 μl 0.2 mg/ml LPS (inject)	Day 7
DEX	5 mg/kg/d dexamethasone (gavage)	Days 1–7
	50 μl 0.2 mg/ml LPS (inject)	Day 7
ATF-L	100 mg/kg/d ATF (gavage)	Days 1–7
	50 μl 0.2 mg/ml LPS (inject)	Day 7
ATF-M	200 mg/kg/d ATF (gavage)	Days 1–7
	50 μl 0.2 mg/ml LPS (inject)	Day 7
ATF-H	1,000 mg/kg/d ATF (gavage)	Days 1–7
	50 μl 0.2 mg/ml LPS (inject)	Day 7

NT: No treatment model group; MC: LPS treatment model group; DEX: Dexamethasone (5 mg/kg) treatment group; ATF-L: ATF 100 mg/kg, ATF-M: ATF 500 mg/kg, ATF-H: ATF 1000 mg/kg.

### Histological examination

Mouse mammary glands were immediately fixed in 4% formaldehyde solution after rinsing the surface stain with PBS. After alcohol dehydration and embedding in paraffin, the tissue was cut into sections of 4 μm thickness, stained with hematoxylin and eosin (H&E) and observed for pathological changes under a microscope (Olympus, Japan).

### MPO and cytokines detection by ELISA

The mammary gland tissues were homogenised in PBS (1,9, w/v). Mouse mammary tissue was rinsed in pre-cooled PBS, dried and weighed, placed in a grinding tube and ground with 9 times the amount of PBS. After grinding, centrifuge for 15 min at 4°C at 2,000 *g* and extract the supernatant as an ELISA test sample and store at −20°C. Equal amounts of 0.5% CTAC were added to the pellet. The tissue samples were again ground for 8 min on a grinder and the homogenate was centrifuged for 10 min at 4°C at 2,000 *g*. The supernatant was taken as a sample for the myeloperoxidase (MPO) assay and stored at −20°C. The manipulation steps of ELISA kit and the MPO kit were according to the manufacturer’s instructions.

### RNA extraction and qRT-PCR analysis

According to the manufacturer’s instructions, total RNA was extracted from mouse mammary glands using TRIzol reagent (GenStar Co., Ltd., China) [Lot#S12FK101], and cDNA was synthesised using the StarScript II First-strand cDNA Synthesis Mix (With gDNA Remover; GenStar Co., Ltd., China) [Lot#S01GA241]. The PCR reactions were performed using RealStar Green Fast Mixture (GenStar Co., Ltd., China) [Lot#S01FK151] on the Light Cycler 96 System (Roche). The mRNA levels were measured by qRT-PCR using a 20 μl reaction system with 3 replicates per sample. GAPDH was used as a housekeeping gene to normalise gene expression and the 2^−ΔΔCt^ comparison method was used to calculate relative mRNA expression levels. The primer sequences (Sangon Biotech Co., Ltd., China) used in this study were shown in Table 2.

**Table 2:** Primers used for qRT-PCR.

Name	Primer sequence (5′–3′)	GenBank accession number	Product size (bp)
TNF-α	CTCATGCACCACCATCAAGG	NM_001278601.1	96
	ACCTGACCACTCTCCCTTTG		
IL1-β	AGCTTCAAATCTCGCAGCAG	XM_006498795.5	72
	TCTCCACAGCCACAATGAGT		
P50	ACAGGGAGATTCGCTGTCAC	XM_006499155.5	75
	CGGTGCCCTCCTTCTTAACC		
IκB	ATGCCAGAACGAGATAGTGAGC	NM_011879.2	156
	AGGTGGCGCAGAAGTAGGT		
CD14	CTCTGTCCTTAAAGCGGCTTAC	NM_009841.4	191
	GTTGCGGAGGTTCAAGATGTT		
TLR4	TCTGGGGAGGCACATCTTCT	XM_036163964.1	110
	AGGTCCAAGTTGCCGTTTCT		
p38	GGCTCGGCACACTGATGAT	NM_001168514.1	214
	TGGGGTTCCAACGAGTCTTAAA		
ERK	AGCCCGTGGAGTAACCCAA	NM_001360542.1	118
	GCATGGAGGGATACATTCCTGT		
Occludin	CTGGATCTATGTACGGCTCACA	NM_001360538.1	129
	TCCACGTAGAGACCAGTACCT		
ZO-1	GAGCGGGCTACCTTACTGAAC	XM_036152895.1	75
	GTCATCTCTTTCCGAGGCATTAG		
Cluadin-1	AGACCTGGATTTGCATCTTGGTG	NM_016674.4	126
	TGCAACATAGGCAGGACAAGAGTTA		
Cluadin-3	ACCAACTGCGTACAAGACGAG	NM_009902.4	78
	CAGAGCCGCCAACAGGAAA		
GAPDH	CAATGTGTCCGTCGTGGATCT	XM_036165840.1	124
	GTCCTCAGTGTAGCCCAAGATG		

### Genomic DNA extraction and 16S-rDNA sequencing of faeces

Mouse faeces were collected under sterile conditions and immediately frozen in liquid nitrogen and stored at −80°C. The genomic DNA of cecal contents was extracted by an E.Z.N.A.^®^ soil DNA Kit (Omega Bio-Tek, Norcross, GA, United States) following the manufacturer’s instruction and detected by 1% agarose gel electrophoresis. The V3-V4 hypervariable regions of the bacterial 16S-rDNA gene were amplified by universal primers 338F (5′-ACTCCTACGGGAGGCAGCAG-3′) and 806R (5′-GGACTACHVGGGTWTCTAAT-3′). The PCR amplification system was 20 μl, and all products were purified by AxyPrep DNA Gel Recovery kit (Axygen, Union City, United States), and quantified by QuantiFluor™ -ST (Promega, United States). According to the standard scheme of Majorbio BioPharm Technology Co., Ltd. (Shanghai, China), PE library was built using TruSeq™ DNA Sample Prep Kit and sequenced by an Illumina Miseq PE300 platform (Illumina, SD, United States). Using UPARSE software (v7.0.1090),^1^ carried out high-quality sequence clustering to eliminate chimeras sequences and obtain Operational Taxonomic Units (OTUs) with 97% similarity. The relationships between samples and species were described using the circos-0.67-7^2^ visualisation method. Alpha-diversity and beta-diversity were analysed according to the abundances of OTUs using the R package. The Wilcoxon rank sum test was used to compare Genus level classifications of bacteria. Linear discriminant analysis (LDA) and LDA effect size (LEfSe) were used to analyse the dominance of the bacterial community between groups.^3^

### Statistical analysis

All data, including figures and tables, were expressed as the mean ± standard deviation (SD) from three independent experiments. Images were generated using GraphPad Prism software (La Jolla, CA, United States). In the animal experiments, mice were randomly grouped, and histological analysis was conducted in a blind manner. One-way ANOVA was used test to analyse the differences between the means of normally distributed data. The results are considered statistically significant at *p* < 0.05 or *p* < 0.01.

## Results

### Ameliorates pathological damage

**Figure 1:**
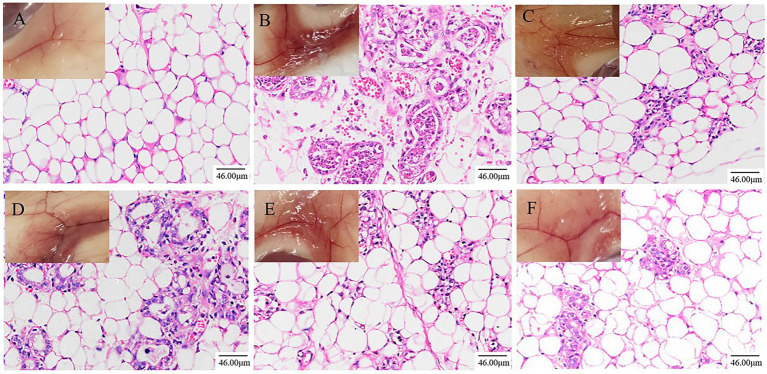
Effects of ATF on pathological changes of LPS-induced mastitis in mice (200×, Scale bar = 46.00 μm). **(A)** NT group; **(B)** MC group; **(C)** DEX group; **(D)** ATF-L (100 mg/kg) group; **(E)** ATF-M (500 mg/kg) group; **(F)** ATF-H (1,000 mg/kg) group.

Pathological sections of mouse mammary tissue were produced and the results of HE staining are shown in Figure 1. The mammary tissue of the NT group of mice and their pathological sections can be seen in Figure 1A. Microscopically, an intact mammary vesicle structure with scattered erythrocytes can be seen, but there are no obvious pathological changes. The mammary tissue of the MC group of mice and their pathological sections can be seen in Figure 1B. Mice with dishevelled coats and poor mental state could be observed, and mammary gland tissue in mice was congested and red. Microscopic examination revealed thickening of the mammary vesicle wall, disruption of the structure of the mammary vesicle and massive infiltration of inflammatory cells and erythrocytes, indicating that the constructed LPS-induced mastitis model in mice was successful. The results after treatment in the DEX group are shown in Figure 1C. The improvement of the LPS-induced erythema is visible to the naked eye, and microscopically, it can be observed that some of the breast structures have been restored and the number of erythrocytes and inflammatory cells in the breast tissue has been reduced substantially. The results after ATF treatment are shown in Figures 1D–F. The degree of engorgement and swelling of mammary tissue in mice gradually decreased with increasing doses. There is a gradual increase in the number of intact breast follicular structures and a gradual decrease in the number of inflammatory cells and erythrocyte infiltration in the pathological sections.

### Effect of ATF on the activity of MPO and secretion of cytokines

**Figure 2:**
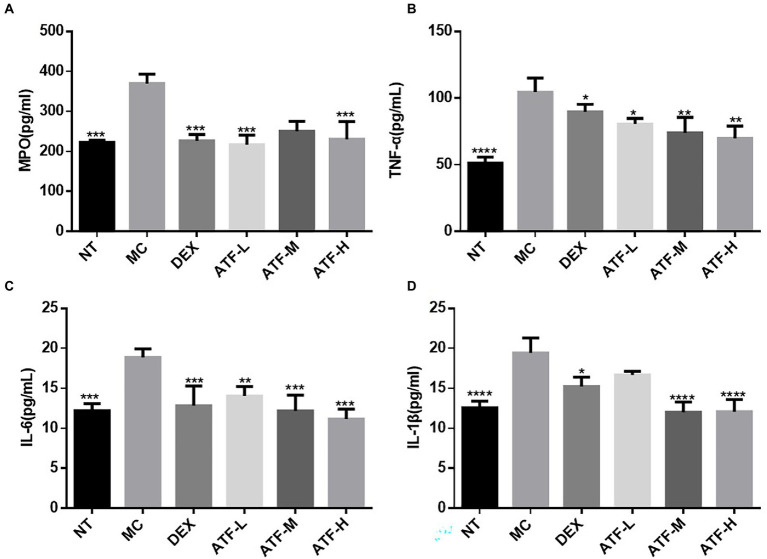
Effect of ATF on MPO activity and inflammatory factor secretion in mouse mammary gland tissue. **(A)** MPO, **(B)** TNF-α, **(C)** IL-6, **(D)** IL-1β. NT: No treatment model group, MC: LPS treatment model group, DEX: Dexamethasone (5 mg/kg) treatment group, ATF-L: ATF 100 mg/kg, ATF-M: ATF 500 mg/kg, ATF-H: ATF 1,000 mg/kg. Data are expressed as mean ± SD, *n* = 5–7. ^*^p < 0.05, ^**^p < 0.01, ^***^p < 0.001, ^****^p < 0.0001 compared with the MC group.

The results of the ELISA kit assay are shown in Figure 2. MPO activity and secretion of pro-inflammatory factors were significantly higher in the MC group than in the NT group, further indicating that the model of LPS-induced mastitis in mice was successfully established. Compared to the MC group, MPO activity and secretion of pro-inflammatory factors were significantly lower in the DEX group, indicating that dexamethasone could reduce LPS-induced inflammation in the mammary glands of mice. ATF was also able to significantly reduce MPO activity and secretion of pro-inflammatory factors. Although not drug-dependent decreasing, the extent of reduction in ATF-M and ATF-H groups did not differ significantly from the DEX group, except for MPO activity.

### Effect on the CD14/TLR4 pathway

**Figure 3:**
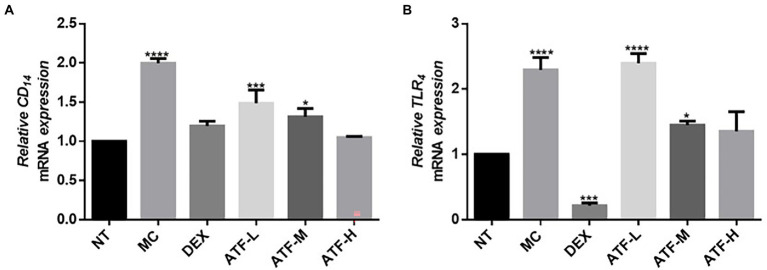
Effect of ATF on mRNA expression of key genes in the CD14/TLR4 pathway. **(A)** CD14, **(B)** TLR4. NT: No treatment model group, MC: LPS treatment model group, DEX: Dexamethasone (5 mg/kg) treatment group, ATF-L: ATF 100 mg/kg, ATF-M: ATF 500 mg/kg, ATF-H: ATF 1000 mg/kg. Data are expressed as mean ± SD, *n* = 5–7. ^*^p < 0.05, ^***^p < 0.001, ^****^p < 0.0001 compared with the NT group.

When LPS invades the organism, CD14 immediately recognizes and binds LPS, which further activates TLR4. Fluorescent quantitative PCR was performed on the mRNA expression of CD14 and TLR4 and the results are shown in Figures 3A,B. Compared to the NT group, there is a highly significant increase in CD14 and TLR4 mRNA expression in the MC group. The mRNA expression of CD14 in the DEX group was not significantly different from that of the NT group, and the mRNA expression of TLR4 was significantly lower than that of the NT group. ATF administration group reduced the mRNA expression of CD14 in a dose-dependent manner, and the ATF-M and ATF-H group were able to reduce the mRNA expression of TLR4.

### Effect on the NF-κB pathway

**Figure 4:**
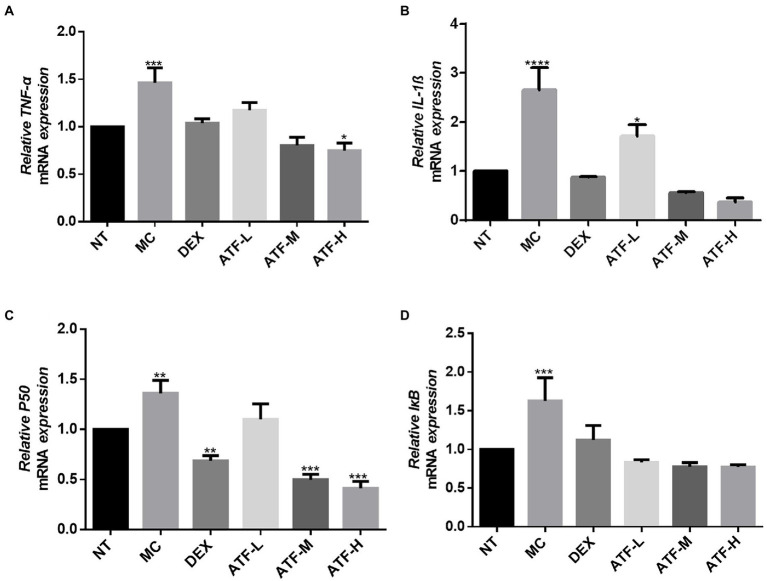
Effect of ATF on mRNA expression of key genes in the NF-κB pathway. **(A)** TNF-α, **(B)** IL-1β, **(C)** P50, **(D)** IκB. NT: No treatment model group, MC: LPS treatment model group, DEX: Dexamethasone (5 mg/kg) treatment group, ATF-L: ATF 100 mg/kg, ATF-M: ATF 500 mg/kg, ATF-H: ATF 1000 mg/kg. Data are expressed as mean ± SD, *n* = 5–7. ^*^p < 0.05, ^**^p < 0.01, ^***^p < 0.001, ^****^p < 0.0001 compared with the NT group.

In the LPS-mediated inflammatory response, the NF-κB signalling pathway is involved in the inflammatory process and plays an important role in the regulation of inflammation (Tang et al., 2021). In the NF-κB pathway, IL-1β, TNF-α, p50 and IκB have pivotal roles, and mRNA expression levels were assessed and detected by qRT-PCR. The results are shown in the Figures 4A–D. The mRNA expression of IL-1β, TNF-α, p50 and IκB was significantly higher in the MC group compared to the NT group. It was shown that LPS was able to cause inflammation in mouse mammary glands by increasing mRNA expression of IL-1β, TNF-α, p50 and IκB in the NF-κB pathway. In the DEX group, the mRNA expression of four important proteins were all significantly decreased compared to the MC group, and the mRNA expression of IL-1β, TNF-α and IκB were not significantly different from that of the NT group. ATF administration group showed a drug-dependent reduction in mRNA expression of IL-1β, TNF-α, p50 and IκB.

### Effect on the MAPK pathway

**Figure 5:**
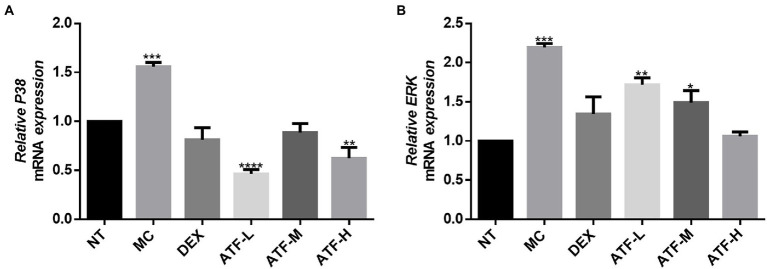
Effect of ATF on mRNA expression of key genes in the MAPK pathway. **(A)** P38, **(B)** ERK. NT: No treatment model group, MC: LPS treatment model group, DEX: Dexamethasone (5 mg/kg) treatment group, ATF-L: ATF 100 mg/kg, ATF-M: ATF 500 mg/kg, ATF-H: ATF 1000 mg/kg. Data are expressed as mean ± SD, *n* = 5–7. ^*^p < 0.05, ^**^p < 0.01, ^***^p < 0.001, ^****^p < 0.0001 compared with the NT group.

MAPK signalling is widespread in cells, and intracellular signalling by CD14 coupled with LPS signalling activates the corresponding NF-κ B and mitogen-activated protein kinase (MAPK) two pathways (Li et al., 2002). The mRNA expression levels of p38 and ERK in the MAPK pathway in mouse mammary tissue were measured by qRT-PCR. The results are shown in Figures 5A,B. The MC group showed a highly significant increase in mRNA expression of p38 and ERK compared to the NT group. The DEX group expressed significantly less compared to the MC group and was not significantly different from the NT group. The mRNA expression of ERK was reduced in a dose-dependent manner in the total flavonoids administration group, while the mRNA expression of p38 was not significantly different in the ATF-M group and the NT group.

### Effect on mRNA expression of tight junction proteins

**Figure 6:**
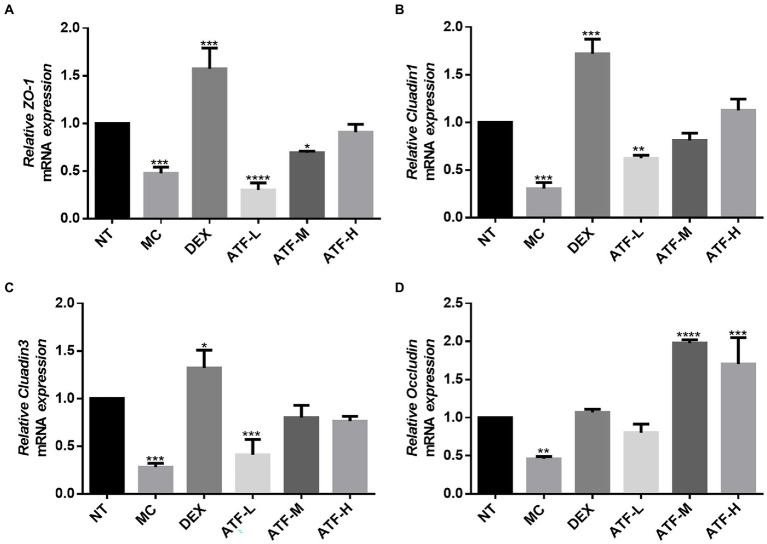
Effect of ATF on mRNA expression of tight junction protein in mouse mammary gland tissue. **(A)** ZO-1, **(B)** Cluadin1, **(C)** Cluadin3, **(D)** Occludin. NT: No treatment model group, MC: LPS treatment model group, DEX: Dexamethasone (5 mg/kg) treatment group, ATF-L: ATF 100 mg/kg, ATF-M: ATF 500 mg/kg, ATF-H: ATF 1000 mg/kg. Data are expressed as mean ± SD, *n* = 5–7. ^*^p < 0.05, ^**^p < 0.01, ^***^p < 0.001, ^****^p < 0.0001 compared with the NT group.

Tight junctions are the main components that make up the blood-milk barrier (Wang et al., 2017). The amount of mRNA expression of tight junction proteins was detected by qRT-PCR in Figure 6. The expression of mRNA for ZO-1, Cluadin1, Cluadin3 and Occludin was significantly reduced in the MC group compared to the NT group. This suggests that LPS injected into mouse mammary tissue inhibits the expression of mRNA for tight junction proteins in mouse mammary tissue. In the DEX group, the mRNA expressions of these four tight junction proteins were significantly increased compared with those in the MC group, especially ZO-1, Cluadin1 and Cluadin3. There was no significant difference in the mRNA expression of ZO-1 and Cluadin3 in the ATF-L group compared to the MC group, but the mRNA expression of Cluadin1 and Occludin was significantly higher than that of the MC group. The mRNA expression of ZO-1 and Cluadin1 was dose-dependent in the ATF-M and ATF-H groups, and there was no significant difference in the mRNA expression of Cluadin3 and Occludin between the two groups. In summary, ATF reduces LPS-induced inflammation in mouse mammary glands by promoting the mRNA expression of tight junction protein.

### Effect on the gut microbiota

**Figure 7:**
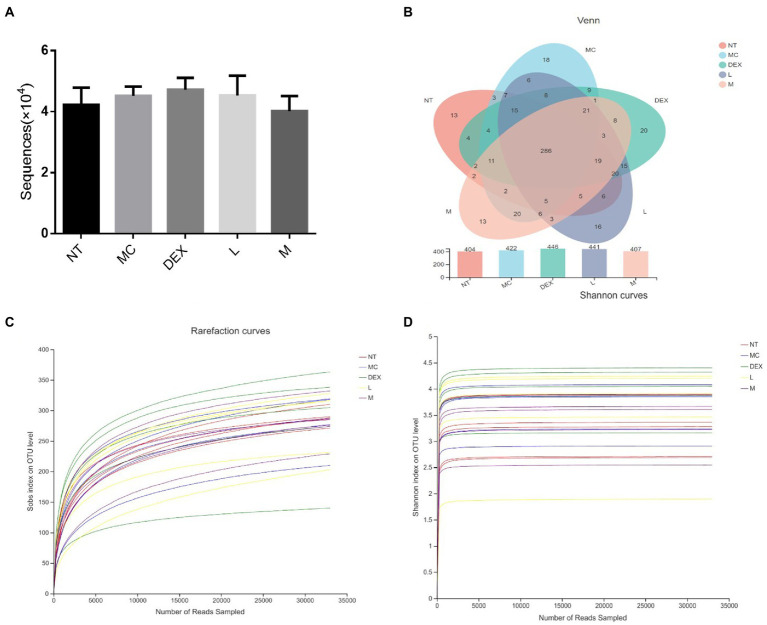
Analysis of the number of DNA sequences in each group and the Venn diagram of species and dilution plots of sobs and Shannon in the mouse gut microbiota. NT: No treatment model group, MC: LPS treatment model group, DEX: Dexamethasone (5 mg/kg) treatment group, L: ATF 100 mg/kg, M: ATF 500 mg/kg.

As shown in Figure 7A, the average number of valid sequences per sample was 32,994 and there were no significant differences in DNA sequences between the groups. A random sampling of sequences was used to construct dilution curves using the number of sequences drawn versus the number they could represent at each taxonomic level. When the curve flattens out, it indicates that the amount of sequencing data is reasonable and that more data will only produce a small number of new species (or OTUs), and vice versa, indicating that more new species are likely to be produced by continued sequencing. The results of the analysis in Figures 7C,D showed that although new systems could be obtained by higher sequencing coverage, most of the gut flora diversity in each sample could be adequately captured at the current sequencing depth. The results of OTU overlap between groups showed in Figure 7B that there were 286 identical OTUs in the 5 groups, with 7, 10, 14, 6 and 3 independent OUTs in the NT, MC, DEX and ATF-L and ATF-M groups, respectively. There was a significant increase in the number of OTUs in the MC group compared to the NT group, and in the DEX group, the increase in the number of OUTs was significantly greater than in the MC group. The number of OUT in the ATF-L group was significantly higher than that of the MC group, which was basically not significantly different from that of the DEX group; the number of OUT in the ATF-M group was basically not significantly different from that of the NT group. OUT data suggest that the diversity of the gut microbiota can be restored by a dose of 50 mg/ml of ATF.

Alpha diversity refers to the diversity within a particular region or ecosystem, and commonly used metrics include Chao, Ace, Shannon and Simpson. Changes in the Chao, ACE, Shannon and Simpson indices based on OTUs can be seen in Table 3. Compared to the NT group, Chao, ACE and Shannon increased in the MC, DEX and ATF-L groups, and Simpson significantly decreased, with the indices of Chao, ACE, Shannon and Simpson in the ATF-M group being closer to those of the NT group. The results showed that ATF-M group were more beneficial to the recovery of the gut microbiota.

**Table 3:** α-diversity indices of gut microbiota in each group.

Groups	Chao	ACE	Shannon	Simpson
NT	350.73 ± 40.12	330.30 ± 14.19	3.51 ± 0.33	0.10 ± 0.024
MC	362.05 ± 25.27	358.26 ± 20.90	3.93 ± 0.12	0.05 ± 0.018^*^
DEX	379.56 ± 40.24	378.90 ± 42.10	4.26 ± 0.19^*^	0.04 ± 0.008^*^
L	378.83 ± 36.46	367.88 ± 18.67	4.12 ± 0.18^*^	0.03 ± 0.010^*^
M	349.11 ± 31.25	337.69 ± 29.38	3.72 ± 0.15	0.07 ± 0.039

NT: No treatment model group; MC: LPS treatment model group; DEX: Dexamethasone (5 mg/kg) treatment group; L: ATF 100 mg/kg, M: ATF 500 mg/kg. Data are expressed as mean ± SD, *n* = 5–7. ^*^p < 0.05 compared with NT group.

**Figure 8:**
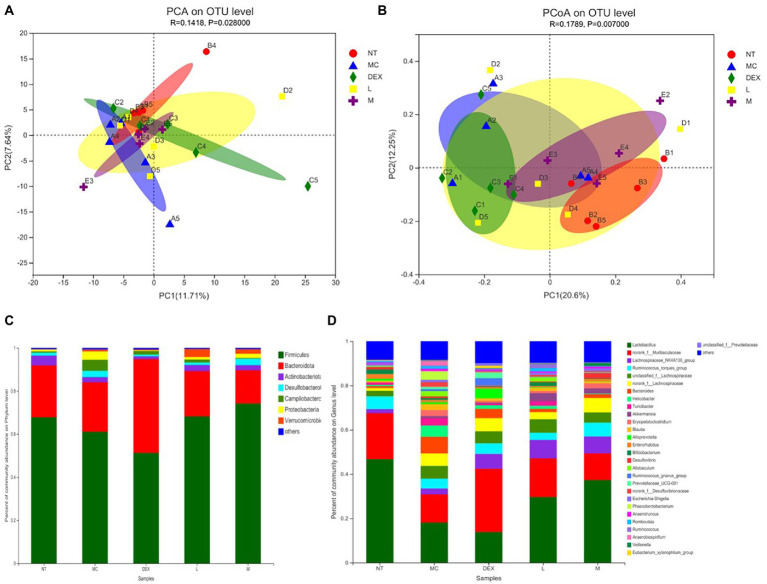
β-diversity analysis **(A,B)** and community composition analysis **(C,D)** of the mouse gut microbiota. NT: No treatment model group; MC: LPS treatment model group; DEX: Dexamethasone (5 mg/kg) treatment group; L: ATF 100 mg/kg, M: ATF 500 mg/kg.

Beta diversity analysis including PCA and PCoA was carried out in order to elucidate changes in microbiota structure between different groups (Chen et al., 2021). PCA uses a table of species (including OTUs) abundance and is based on direct mapping of Euclidean distances, while PCoA is based on a matrix of selected distances, both of which are used to identify potential principal components influencing differences in the composition of the sample communities through dimensionality reduction (Li et al., 2021). The more similar the species composition of the sample, the closer it is reflected in the PCA plot. As shown in Figures 8A,B, although there was a small amount of overlap in all five groups, the ATF-M group was closer to the NT group than the MC group. The results of the analysis of the community composition of the gut microbiota of mice in the NT group, the MC group, the DEX group and the ATF-L and ATF-M groups are shown in Figures 8C,D. At the phylum level in Figure 8C, Firmicutes and Bacteroidota are the two most dominant phyla, accounting for nearly 90% of the total number of bacteria. Compared to the MC group, there was a significant increase in Proteobacteria, Campilobacterota and Desulfobacterota in the MC group and Bacteroidota in the DEX group. The proportion of Firmicutes and Bacteroidota was close to that of the NT group in the ATF-L and ATF-M groups, but Verrucomicrobiota was significantly higher than that of the NT group. To further explore differences in the gut microbiota of certain taxa, several dominant microorganisms at the genus level were analysed and compared. At the genus level in Figure 8D, Lactobacillus and norank_ f_ Muribaculaceae were the two genera that occupied the major dominance. Compared to the NT group, the proportion of the MC group occupied by Lactobacillus and norank_f_Muribaculaceae was significantly lower overall, with significant increases in unclassified_f_Lachnospiraceae, norank_f_Lachnospiraceae, Bacteroides and Helicobacter. The proportion of the genus Lactobacillus and norank_f_Muribaculaceae in the DEX, ATF-L and ATF-M group was significantly increased compared to the MC group. But compared to the NT and MC groups, there was a significant increase in Lachnospiraceae_NK4A136_group and a decrease in Bacteroides.

**Figure 9:**
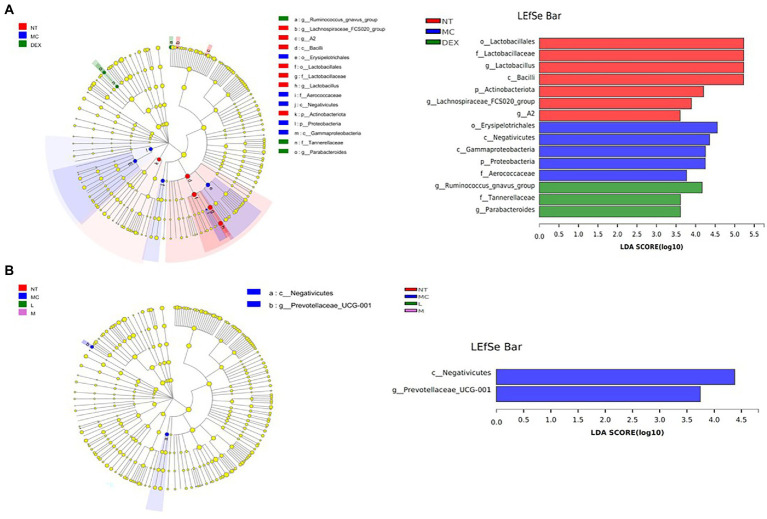
Results of the LEfSe analysis of the gut microbiota of mice. **(A)** NT group, MC group and DEX group, **(B)** NT group, MC group, ATF-L group and ATF-M group. NT: No treatment model group; MC: LPS treatment model group; DEX: Dexamethasone (5 mg/kg) treatment group; L: ATF 100 mg/kg, M: ATF 500 mg/kg.

LEfSe was used to analyse key system types of intestinal flora in different groups, as shown in Figures 9A,B. In a comparison of the NT, MC and DEX groups in Figure 9A, it was found that o_u Lactobacillales, f_u Lactobacillaceae, g_u Lactobacillus, c_u Bacilli had a higher LDA score in the NT group, o__Erysipelotrichales, c__Negativicutes, c__Gammaproteobacteria, p__Proteobacteria had higher LDA scores in the MC group. The key bacterial types affecting the imbalance of the gut microbiota in the MC group were o__Erysipelotrichales and c__Negativicutes. In the DEX group, LDA scores were higher for g__Ruminococcus_gnavus_group, f_Tannerellaceae and g_Parabacteroides. It has been shown that Ruminococcus gnavus can induce inflammatory bowel disease (Henke et al., 2021). This suggested that dexamethasone, although able to reduce the inflammation of LPS-induced mastitis in mice, may cause intestinal disease in mice. The results in Figure 9B show that when the NT, MC and ATF groups were analysed and compared, species differences were mainly found in the MC group, with a significant effect of higher LDA scores for the major c__Negativicutes.

## Discussion

In modern pharmacological studies, it has been shown that *A. cantoniensis* has hepatoprotective, choleretic, antibacterial, anti-inflammatory, immune enhancing, free radical scavenging, regulating smooth muscle function and improving endurance (Yang et al., 2015). Mastitis is one of the diseases that currently affect dairy farming more than any other. *E. coli* is one of the most common pathogens among environmental contaminants causing clinical forms of mastitis in dairy cows (Hulsen, 2000). It has been shown that LPS is one of the known inflammatory factors in Gram-negative infectious diseases such as *E. coli* mastitis (Li et al., 2017; Chu et al., 2019), and the effective control of inflammation in the treatment of such diseases is an important issue in veterinary clinical practice. When LPS invades murine mammary tissue, it causes damage to the mammary tissue, causing sloughing of acinar epithelial cells, changes in shape and size, accompanied by massive infiltration of inflammatory cells and engorgement of mammary tissue (Ou, 2020). In severe cases, the blood-milk barrier can be broken, leading to blood entering the mammary glands, which can be accompanied by blood in the milk and a marked increase in redness and swelling of the mammary glands, which can lead to the death of the cow in severe cases (Xu et al., 2018). This could explain the pink, milky discharge that appears at the teat site in some females 12 h after LPS is injected into the mammary ducts of mice. In this study, we found that after the mouse mammary tissue was injected with LPS, the mouse’s papilla appeared red and swollen macroscopically. Inflammatory cells and erythrocytes infiltrating the mammary tissue, thickening of the glandular vesicle wall and disorganised glandular vesicle structure could be observed in pathological sections, indicating that the mouse mastitis model has been successfully established. Gavage of high doses of dandelion sterols resulted in significant improvement in mammary gland epithelial cell detachment and tissue haemorrhage in rat mammary gland tissue (San, 2014). Gavage of ethanolic extract of Chinese propolis significantly reduced the pathological damage caused by LPS-induced destruction of glandular vesicle structure and inflammatory cell infiltration in mammary gland tissue of mice (Song et al., 2021). In this study, it was found that gavage of the total flavonoid solution of *A. cantoniensis* was also able to reduce LPS-induced damage to the mammary tissue of mice and reduce the infiltration of inflammatory cells and red blood cells. In this study, the mRNA expression of ZO-1, Cluadin1, Cluadin3 and Occludin was significantly reduced in the mammary tissues of the MC group by real-time fluorescence quantitative PCR, indicating that LPS invades the body and damages the blood-milk barrier while inhibiting the mRNA expression of tight junction proteins, causing mastitis in mice. Administration of dexamethasone or ATF to mice promoted the mRNA expression of four tight junction proteins, ZO-1, Cluadin1, Cluadin3 and Occludin, after LPS damaged the blood-milk barrier of the mammary gland, which promoted the repair of the blood-milk barrier and reduced the inflammation of the mammary gland induced by LPS.

MPO is a haemoglobin protein rich in neutrophils, synthesised in the bone marrow by granulocytes before they enter the circulation and stored in *Aspergillus* granules (Thaysen-Andersen et al., 2015). External stimuli can lead to the aggregation of neutrophils, resulting in the release of MPO (Parlar et al., 2021). ATF can reduce MPO activity in mammary tissue of mastitis mice, and it can reduce the aggregation of neutrophils to alleviate LPS-induced inflammation in the mammary gland of mice. As key messenger molecules in the exchange of information between cells, cytokines are primarily involved in the regulation of immune homeostasis and inflammatory responses (Murtaugh and Foss, 2002). In mastitis, immune cell activity is mainly regulated by the pro-inflammatory cytokines TNF-α, IL-1β and IL-6, which are typically pro-inflammatory cytokines (Alluwaimi, 2004). In this study, ATF significantly reduced TNF-α, IL-1β and IL-6, which were elevated by the injection of LPS into the mammary tissue, and reduced the secretion of pro-inflammatory factors to reduce inflammation in the mammary gland of mice. When LPS invades the organism, the lipopolysaccharide-binding protein LBP immediately recognises and binds LPS, while binding to CD14 on the surface of myeloid-derived cells, forming the LPS-LBP-CD14 triplet complex, which will later bind to TLR4 with the help of MD-2, activating TLR4 and causing it to dimerise (Peri et al., 2010). After TLR4 is activated, the intracellular group conformation changes, and after transmitting the signal into the cell, it will further activate MAPK and NF-κB signalling pathways, eventually causing the release of IL-1, IL-6, TNF-α, NO, etc., resulting in an inflammatory response (Seo and Jeong, 2020). NF-κB is a nuclear transcription factor that plays an important role in the inflammatory response (Wu et al., 2018). In this study, qRT-PCR was used to detect the expression of mRNAs such as CD14, TLR4, IL-1β, TNF-α, p50, IκB, p38 and ERK. LPS was found to cause inflammation in mouse mammary glands by upregulating mRNA expression of the CD14/TLR4/NF-κB/MAPK pathway, while dexamethasone and ATF reduced LPS-induced mastitis in mice by downregulating mRNA expression of this pathway.

Under normal conditions, the gut microbiota maintains a symbiotic or antagonistic relationship with each other, which is the main reason why the host gut microbiota is in micro-ecological balance (Zhen et al., 2017; Liu, 2018). Once the gut microbiota is disordered, it will cause an abnormality in the body’s health condition. After LPS was injected into the milk ducts of mice in this study, the composition of the gut microbiota was altered in mastitis-stricken mice compared to normal mice. The main increase was in Campilobacterota and Proteobacteria. It has been found that Campylobacter can cause food poisoning in the form of severe enteritis (inflammation of the small intestine; Davalos et al., 1981); the bacteria of the phylum Aspergillus are all Gram-negative and their outer membrane consists mainly of LPS. After the administration of dexamethasone in the DEX group, there was a significant decrease in the thick-walled phylum and instead a significant increase in the Anaphylae phylum in the intestinal flora of mice with mastitis, and another significant change in the Actinomycete phylum. Actinomyces is a filamentous Gram-positive bacterium with a higher GC content compared to another group of Gram-positive bacteria in the gut microbiota, Firmicutes (Stopinska et al., 2021). One of the most striking features of Actinomycetes has been found to be their ability to produce large numbers of a wide variety of antibiotics (Li et al., 2008). A decrease in the proportion of the Actinobacteria phylum may therefore affect the homeostasis of the organism’s intestinal microbiota. At the genus level, Lactobacillus (0.467) and norank_f_Muribaculaceae (0.208) were the dominant genera in normal mice. Lactobacillus is an important genus of Firmicutes. Lactobacillus is a Gram-staining positive, non-spore-forming bacterium with the ability to regulate intestinal flora, enhance immunity, protect the gastric mucosa, improve intestinal function, laxative, anti-diarrhoea, promote digestion, anti-tumour, antioxidant and other effects (Huang et al., 2017). A direct reduction of Lactobacillus to 0.181 and of norank_f_Muribaculaceae to 0.127 in the intestinal flora of mice with mastitis was found, with a disturbance in homeostasis, which could indicate that LPS-induced mastitis in mice leads to alterations in the gut microbiota of mice as well. Treatment of mastitis in mice by dexamethasone gavage in the DEX group revealed that Lactobacillus in the intestinal flora of mice would continue to decrease to 0.139. Norank_f_Muribaculaceae increased and exceeded the NT group, the results suggest that dexamethasone does not achieve restoration of the intestinal microbiota of mice in alleviating LPS-induced mastitis in mice. ATF administration group found that the genus Lactobacillus increased with increasing dose and the ratio of Lactobacillus to norank_f_Muribaculaceae (0.591 in the ATF-L group and 0.332 in the ATF-M group) was closer to that of the NT group (0.445) and significantly smaller than that of the MC group (0.702) and the DEX group (2.058). In this study, there was a significant increase in Akkermansia compared to the other three groups in the ATF-L and ATF-M groups, which is in line with the results that Gegen Qinlian Decoction (GQD) can significantly increase the amount of Akkermansia (Li et al., 2021). Akkermansia is an oval-shaped Gram-negative anaerobic bacterium that clings to the intestinal mucosa, where it protects the intestine from pathogens through competitive action (Gao et al., 2018). The infusion of two herbs, ATF and Gegen Qinlian Decoction (GQD), can increase the amount of Akkermansia, which may provide a new direction of thinking to effectively improve the immunity of the body and enhance the anti-inflammatory effect.

In the analysis of the key phylotypes of the gut microbiota in the different groups using LEfSe, it was found that when the positive drug dexamethasone was used to treat LPS-induced mastitis in mice, significant differences were still found in the intestinal flora of the mice compared to normal mice. In the treatment of LPS-induced mastitis using ATF, differences were found in the intestinal flora of mice compared to normal mice and mice suffering from mastitis, with differences only in c__Negativicutes and g__Prevotellaceae_UCG-001 in the MC group. One study found an increase in the abundance of intestinal flora with dexamethasone (Song et al., 2020), which is consistent with the findings in this study that treatment with dexamethasone resulted in an increase in the abundance of intestinal flora in mice (Chao, ACE and Shannon indices were all higher than in the normal group of mice). Studies have found that NSAIDs cause changes in microbial populations and lead to intestinal damage (Dai et al., 2020). The above studies have shown that the use of the anti-inflammatory drug dexamethasone, while reducing the inflammatory response in the mammary tissue of mice induced by LPS, can further affect the homeostasis of the intestinal flora and can even lead to other serious consequences. This inflammatory response can also be reduced by the ATF use, although the therapeutic effect still differs from that of the anti-inflammatory drug dexamethasone. However, the advantage is that it improves the disruption of intestinal flora in mice caused by this inflammation and achieves a green effect without antibiotic residues.

## Conclusion

ATF can reduce the pathological damage of LPS-induced mastitis in mice, decrease MPO activity and inflammatory factor secretion in mammary tissue and serum, inhibit mRNA expression of key genes in the CD14/TLR4/NF-κB/MAPK signalling pathway and promote mRNA expression of tight junction proteins in the blood-milk barrier, effectively improving the inflammatory response of LPS-induced mammary tissue in mice. At the same time, ATF can improve the disturbance of the intestinal microbiota caused by LPS, regulate the relative abundance of the dominant flora and restore the diversity of the gut microbiota, which is distinct from the anti-inflammatory drug dexamethasone.

## Data availability statement

The datasets presented in this study can be found in online repositories. The names of the repository/repositories and accession number(s) can be found at: https://www.ncbi.nlm.nih.gov/, PRJNA799400.

## Ethics statement

The animal study was reviewed and approved by Committee of Guangxi University (Nanning, China).

## Author contributions

W-JS, E-YW, and Q-ZL conceived the study and designed the project. E-YW, G-YZ, B-CX, X-GC, K-YH, YW, and L-ZH performed the experiment. W-JS, E-YW, and G-YZ analysed the data and drafted the manuscript. E-YW, B-CX, and X-GC sorted out the charts. All authors contributed to the article and approved the submitted version.

## Funding

Funding was provided by the Key Research and Development Plan of Guangxi, China (AB19245037), the Major R&D Project of Nanning (20212138), Natural National Science Foundation of China (31760746), TCM Industrial Pioneers (Gui Nong Ke Meng 202211), and the Research Initiation Project for High-level Talents of Yulin Normal University (G2022ZK05).

## Conflict of interest

The authors declare that the research was conducted in the absence of any commercial or financial relationships that could be construed as a potential conflict of interest.

## Publisher’s note

All claims expressed in this article are solely those of the authors and do not necessarily represent those of their affiliated organizations, or those of the publisher, the editors and the reviewers. Any product that may be evaluated in this article, or claim that may be made by its manufacturer, is not guaranteed or endorsed by the publisher.

## Footnotes

^1^ http://drive5.com/uparse/

^2^ http://circos.ca/

^3^ http://huttenhower.sph.harvard.edu/galaxy/root?tool_id=lefse_upload
